# Seeking Protection in the Heart of the Storm: Findings from a Grounded Theory Study

**DOI:** 10.1155/2024/6185455

**Published:** 2024-07-30

**Authors:** Mehraban Shahmari, Nahid Dehghan Nayeri, Alvisa Palese, Seemin Dashti, Arpi Manookian

**Affiliations:** ^1^ Department of Medical-Surgical School of Nursing and Midwifery Ardabil University of Medical Sciences, Ardabil, Iran; ^2^ USERN Care (TUMS) Office School of Nursing and Midwifery Tehran University of Medical Sciences, Tehran, Iran; ^3^ Nursing and Midwifery Care Research Center School of Nursing and Midwifery Tehran University of Medical Sciences, Tehran, Iran; ^4^ Department of Medicine University of Udine, Udine, Italy; ^5^ Department of Health Education and Promotion Faculty of Health Tabriz University of Medical Sciences, Tabriz, Iran; ^6^ Department of Nursing Ardabil Branch Islamic Azad University, Ardabil, Iran; ^7^ Department of Medical-Surgical Nursing School of Nursing and Midwifery Tehran University of Medical Sciences, Tehran, Iran

## Abstract

**Background:**

Nurse protection is a multifaceted concept that has become increasingly relevant in recent years. Despite its importance in effectively managing pandemics, there is still a gap in knowledge about how nurses achieve protection in hospitals.

**Objective:**

To describe the process of seeking protection among nurses during the COVID-19 pandemic.

**Methods:**

A grounded theory approach from 2020 to 2022, employing purposive and theoretical sampling. Face-to-face and online interviews were conducted with 25 participants, resulting in 29 interviews. Data analysis was carried out using Corbin and Strauss's method (2015).

**Results:**

The analysis revealed that nurses encountered numerous obstacles related to patients, nurses themselves, organizations, and the passage of time during the COVID-19 pandemic. These challenges were intertwined with three key concepts: transformations, inequalities, and emotional challenges, highlighting the multifaceted nature of nurses' protection concerns. In response, nurses employed a protective strategy bolstered by catalysts to address these challenges. This strategy encompassed both optimistic outlooks (“Bright horizon”) and somber reflections (“Unpleasant reflection”). Ultimately, seeking protection in the heart of the storm emerged as the core concept, representing the multifaceted process through which nurses navigate and seek protection amidst the unique challenges posed by the pandemic.

**Conclusions:**

This study presents a comprehensive theory that explicitly explains the multifaceted process of seeking protection among hospital-employed nurses during a pandemic. The theory captures the interconnectedness of challenges faced by nurses and the protective strategies they employ while acknowledging the nuanced balance between hopeful prospects and sobering reflections. *Implications for Nursing Management*. Policymakers, managers, and educators can utilize the findings to improve nursing management and support systems. By increasing awareness, addressing challenges, and providing robust support, they can enhance the well-being and effectiveness of nurses during healthcare crises, ultimately improving patient care quality.

## 1. Introduction

The COVID-19 pandemic presented significant challenges to healthcare systems globally, especially affecting nurses who were at the forefront of patient care [1]. Nurses faced extreme working conditions and threats to their protection and safety while caring for COVID-19 patients [2, 3]. They were exposed to both physical and psychological risks, resulting in infections up to deaths among healthcare workers [2–4].

Ensuring the protection and safety of nurses during a pandemic is crucial due to their vital role in managing infectious diseases and protecting public health [3]. In this study, the focus is on the protection and safety of nurses themselves, distinct from the concept of patient safety, which is also of paramount importance. The term “protection” refers to the measures and strategies nurses employ to defend themselves against the various threats and hazards they face, such as exposure to the virus, inadequate personal protective equipment (PPE), and high-stress levels.

Various theoretical perspectives, such as needs theory, managerialism, and motivational theory, have recognized the importance of different types of safety, including physical, psychological, professional, and social safety. These theories provide valuable insights into the multifaceted nature of safety. For example, Maslow's hierarchy of needs positions physical safety as the second fundamental requirement, emphasizing its significance [5]. Herzberg's theory distinguishes between hygiene factors and motivators about professional safety, encompassing elements like job security, adequate staffing, and growth opportunities [6]. The Job Demands-Resources (JD-R) model highlights the balance between demands and resources to ensure psychological safety, taking into account factors like heavy workloads and emotional stress [7]. Theoretical frameworks like social support theory or the JD-R model shed light on how social interactions impact the overall sense of safety and well-being [8]. By incorporating these theories, we can develop a comprehensive understanding of the importance of safety and its diverse dimensions for nurses during the COVID-19 pandemic. According to these theories, we can also better understand what happens when safety is threatened: nurses compromised safety can adversely affect their health, work quality, and performances, leading to dissatisfaction, increased absenteeism, and intentions to leave the profession [1, 3]. These factors ultimately result in poorer patient care and increased pressure on hospitals and healthcare costs [3, 4].

Given the unprecedented nature of the pandemic, prioritizing healthcare worker protection and safety was paramount to ensure effective patient care management [9]; in this context, learning from the experience lived by deepening nurses' perceptions, behaviors, and interactions related to staff protection and safety is crucial [10]. Therefore, this study aims to shed new light on the concept of “seeking protection” as perceived by nurses. Nurses seek protection and safety not only as part of their responsibility of caring for patients affected by the COVID-19 infection but also from the various risks associated with their frontline duties, including exposure to the virus, PPE, and high-stress levels. Operationalizing this concept may inform how to mitigate these risks to protect nurses' well-being while they fulfill their critical role during a pandemic. Consequently, qualitative research can uncover nurses' experiences, feelings, and deeper perspectives, providing valuable insights to support and prepare healthcare professionals and improve organizational actions in future pandemics.

Available evidence suggests that seeking protection and safety among nurses is an interactive process rooted in a specific natural context and reflects the group's nursing-related and social interactions within a real work environment [11, 12]. This process can be influenced by the culture, norms, routines, and habits of each context. In particular, the unique characteristics of the context in Iran, such as the low nurse-to-patient ratio [13], lack of experience in dealing with a widespread pandemic, challenging economic conditions, and insufficient resources to combat COVID-19 [14], make it even more crucial to provide comprehensive information and gain a deep understanding of the process. Despite numerous research studies, the literature still indicates a knowledge gap in this area [15].

Therefore, this study aims to elucidate the multifaceted process of seeking protection among hospital nurses amidst the unique challenges posed by the COVID-19 pandemic, particularly in contexts with specific socioeconomic and healthcare system characteristics, such as the Iranian country.

## 2. Methods

### 2.1. Study Design and Setting

A qualitative study was conducted using the grounded theory [16–18]. Grounded theory aims to explore, understand, and describe psychological and social processes within natural social settings. By adopting this approach, the researchers immersed themselves in the participants' world to gain insights and contribute to acquiring their experiential knowledge [19, 20]. Therefore, grounded theory was chosen to develop a theory from experiential data [20]. The methodology version proposed by Corbin and Strauss in 2015 was followed for its structured data analysis system, which is especially suitable for novice researchers [20, 21]. The study adhered to the Standards for Reporting Qualitative Research (SRQR) [22] (see Table 1 in the Supplementary file) and involved academic medical centers in two Iranian cities with populations of 13,267,637 and 1,270,420, respectively, between 2020 and 2022.

### 2.2. Study Participants

Researchers identified participants among nurses working in academic medical centers designated to care for COVID-19-positive patients using purposive sampling. The inclusion criteria included nurses educated at the level of Bachelor of Science in Nursing (BSN), which is the minimum nursing qualification in Iran. Additionally, participants needed to demonstrate an interest in communication and expressing their views and experiences, as this was considered essential for providing valuable insights. Furthermore, participants were selected based on their experience in caring for COVID-19 patients or working in facilities designated to receive such patients, ensuring firsthand knowledge and experiences, and thus helping in understanding the challenges faced in this context. These criteria were crucial in achieving the maximum theoretical richness and in-depth understanding of the experience lived by nurses [20]. Moreover, to achieve maximum theoretical variations, richness, and diversity, the researchers identified participants with diverse personal and professional characteristics to obtain comprehensive data and develop findings with higher transferability. After the first interview, additional interviews were conducted based on the analysis of previous interviews and using a theoretical sampling [20]. For example, when the topic of “unsafety of non-COVID-19 wards” was developed, the first researcher felt the need to interview nurses who did not work in COVID-19 wards as the next step, which was agreed upon by a second researcher. Theoretical sampling continued until saturation was reached, as judged by the research team [20]. No friendship was established between the interviewer and the participants before the interview. A total of 27 nurses were contacted consecutively, and all agreed to be interviewed except for two who refused. The demographic characteristics of the participants are provided in Table 1.

### 2.3. Data Collection

A female researcher (MSh), educated at the doctoral level and holding a faculty position, conducted audio-recorded interviews with participants' consent and collected field notes when necessary. The interviews started with an open-ended question: “Given your experience caring for COVID-19 patients (or during the pandemic for those not in COVID-19 wards, see Table 1), how did you protect yourself?” As data collection progressed, interviews followed a semistructured format, with questions like: “What influenced your actions?” After theoretical sampling, when some issues emerged as “inadequate support, discrimination, and dissatisfaction with PPE rationing,” interviews were then conducted with a head nurse and a supervisor responsible for coordinating shifts and PPE in COVID-19 wards. They were asked about challenges faced during the pandemic and strategies used to ensure nurses' safety and protection. Subsequent interviews were guided by participant responses, with exploratory questions, such as: “Can you explain further?” and “Why?” Participants were given the chance to add to their comments at the end of each interview. Of the 29 interviews with 25 participants, 22 were conducted in person while adhering to protective protocols and seven were conducted by online video call via WhatsApp at the request of the participants. In these cases, four supplemental interviews were conducted to ensure the richness and depth of the interviews and to confirm the findings. The average, minimum, and maximum duration of the interviews were 50, 45, and 90 minutes, respectively, reflecting variations in the duration spent with each participant.

### 2.4. Data Analysis

The data analysis in the grounded theory method is a dynamic and circular process carried out through continuous and simultaneous comparison, data collection, and analysis, leading to the extraction of deep and reliable information about the phenomenon of interest [20]. In our study, the researchers used the method of Corbin and Strauss (2015) [16] to discover the basic theory, employing open coding to identify concepts, developing concepts in terms of their properties and dimensions, analyzing data for context, bringing the process into the analysis, and integrating categories to identify the core category [20].

The MAXQDA10 software was used to manage the large dataset, which consisted of about 580 verbatim-transcribed pages. Initially, one of the researchers (MSh) listened to the interviews, transcribed them, and reread the text and notes multiple times. The data were then segmented into manageable chunks, and semantic units were created.

Initial categories were formed through brainstorming and comparing concepts, with constant comparisons to classify codes based on similarities and differences. The researchers carefully considered both micro- and macroconditions affecting nurses' safety. Memos and diagrams were used from the beginning of the analysis to aid in this process. By analyzing data and reviewing narratives repeatedly, the researchers identified the interactions, feelings, strategies, and behaviors of participants. “Seeking protection in the heart of the storm” emerged as the core category, encompassing the experiences of all participants. Finally, the researchers examined the relationship between concepts and categories, developing a theory to explain the phenomenon under study. An example of the process of forming a main category is shown in Table 2 in the Supplementary file.

### 2.5. Trustworthiness and Rigor

To ensure the quality of our study, we applied the criteria proposed by Corbin and Strauss (2015), which include checkpoints for assessing the methodological consistency and the quality and applicability of a grounded theory-based study [20]. Additionally, we incorporated the criteria of credibility, dependability, confirmability, transferability, and authenticity as suggested by Guba and Lincoln [23]. Specifically, data were collected simultaneously in an iterative process, and actions and processes were analyzed and conceptualized rather than focusing on themes and structures. Comparative methods, such as constant comparison, were used to confirm or reject codes and categories, determine relationships between categories, and uncover hidden processes in the data. Inductive categories were developed through a constructive approach, and theoretical sampling was employed to identify variations in categories. We ensured prolonged interactions with participants, allocated sufficient time for data collection, maintained trustworthy communication, and involved member reviews. The research team reviewed extracted codes, compared themes, conducted discussions to reach consensus on issues, peer-reviewed the data, and preserved preliminary study documents. We also provided a detailed description of the research process, participant characteristics, and research context and selected diverse participants to ensure the applicability of the data to other similarly structured communities.

### 2.6. Ethical Considerations

The Ethics Committee of Tehran University of Medical Sciences (TUMS) granted ethical approval (IR.TUMS.FNM.REC.1399.193). All participants provided consent after receiving explanations about the study's objectives, their voluntary participation, and their freedom to withdraw at any point. They were also given the option to choose the interview time and location. Permission was secured for audio-recording the interviews. Participants were ensured regarding data confidentiality, including the deletion of audio files after the study and anonymization of all participants and working units in documents (e.g., Participant 1, P1). Subsequently, the audio files underwent verbatim transcription and anonymization.

## 3. Findings

### 3.1. Emerging Categories and Subcategories

The analysis of data gathered from participants uncovered a range of challenges that hospital nurses encountered during the COVID-19 pandemic. These obstacles encompassed various aspects, such as patient-related issues (e.g., increased patient load, poor patient compliance), challenges faced by nurses themselves (e.g., working conditions), organizational factors (e.g., human resource difficulties, inadequate protective measures), and the passage of time (e.g., from extreme reactions to normalization).

These conditions were found to be interconnected and gave rise to three key concepts: transformations, inequalities, and emotional challenges. These concepts reflected the complex and multifaceted nature of the issues faced by nurses in their efforts to ensure their protection and safety. In response to these challenges, nurses adopted a strategy of seeking protection, which was fueled by catalysts that propelled them in this pursuit.

**Figure 1:**
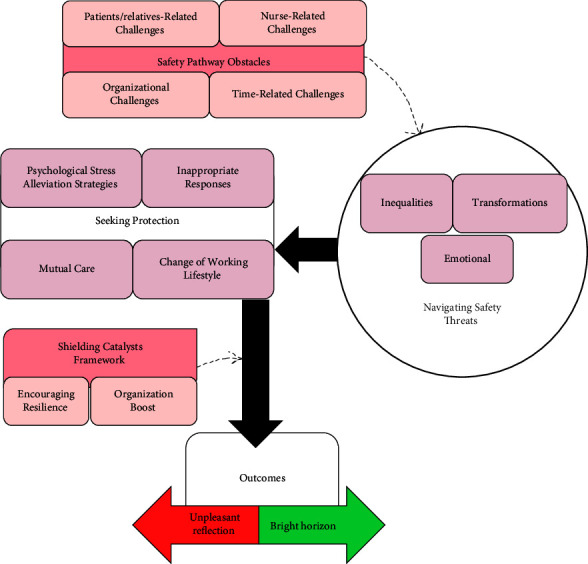
The structure and the process of the “seeking protection in the heart of the storm” theory.

The strategy of seeking protection exhibited both optimistic prospects, symbolized by a “Bright horizon,” and somber reflections, represented by an “Unpleasant reflection.” These contrasting elements shaped nurses' approach to safeguarding themselves during the pandemic. Consequently, the core concept that emerged from this analysis was termed “seeking protection in the heart of the storm” as depicted in Figure 1 and elaborated upon in detail in Table 2.

#### 3.1.1. Contextual Conditions

Participants emphasized the paramount importance of understanding the dynamics of safety pathways, including obstacles and catalysts, in shaping the journey toward safety and protection for hospital nurses during the COVID-19 pandemic. These contextual conditions consist of two main categories and six subcategories (refer to Table 2 and Figure 1), which have been identified as crucial in influencing the seeking protection process among frontline nurses. This in turn contributes significantly to their well-being and the effectiveness of patient care.

*(1) Safety Pathway Obstacles*. Participants indicated that they encountered a spectrum of impediments along the safety pathway categorized into four main challenges: organizational, patient/relative-related, time-related, and nurse-related challenges.

*Organizational Challenges*. The challenges faced by organizations have triggered several issues for nurses: staffing problems, difficulties in information gathering, in providing training; unfavorable infrastructure factors, time-consuming documentation for accreditation, and inadequate supervision, support, and organizational assistance were all reported. Additionally, varying ward-related safety conditions were shared.

*“In the first few days, we were suddenly sent to the COVID-19 intensive care unit without any training or preparation; we were confused. We did not know what was going to happen. It scared us, and many of my colleagues caught the virus.” (P 7)*

Nurses shared their personal experiences with safety hazards related to the shortage and poor quality of PPE.

*“The protective equipment was scarce and of poor quality. In the first few months, they gave us two N95 masks and told us to wash and reuse them.” (P 17)*

Participants disclosed that organizational challenges led to various physical, psychological, and professional issues.

*Patient/Relative-Related Challenges*. According to participants, another factor impacting nurses' safety and protection was dealing with patients and their relatives. This included ensuring patient care in challenging conditions, inadequate patient compliance, noncompliance among relatives/visitors, and high expectations from patients' relatives regarding nursing care.

*“In intubated patients, the stress is even greater, especially during resuscitation or suctioning. The cardiopulmonary resuscitation team, which is more directly involved, told us that the risk of infection increases when a bag valve mask is administered manually.” (P 10)*

Regarding safety risks related to noncompliant visitors, one nurse reported:

*“Companions and visitors are fully or partially non-compliant on the ward. For example, they don't use masks or just put them over their mouths. No matter how strictly we follow the rules, their non-compliant behavior puts us at risk and increases our fear and anxiety.” (P 21)*

Another nurse emphasized the safety threat posed by relatives in response to unexpected outcomes.

*“We often faced situations where patients arrived late, worsening their condition. For instance, they were brought in when their lungs were already inflamed. If such patients were then connected to life support machines and unfortunately passed away, their relatives would direct aggression towards us, blaming us for the outcome.” (P 16)*

*Time-Related Challenges*. The passage of time has had a significant impact on working conditions, the availability of PPE, and the responses of caregivers during the early and later phases of the pandemic. Time has played a role in how nurses achieve safety, with changes in their psychological reactions influencing their adherence to safety protocols. Initially, nurses experienced intense anxiety and fear, leading to strict adherence to protocols. However, as time passed, they began to return to a sense of normalcy.

*“In the beginning, we had difficulties with the lack of protective equipment or inferior masks. In the first few months, they gave us a single N95 mask for a week and told us to wash and reuse it, but in the beginning, we adhered more strictly to protocols out of deep fear and anxiety. Over time, however, our sensitivity weakened, and some colleagues no longer took the disease seriously.” (P 21)*

*Nurse-Related Challenges*. According to participants, nurse-related factors directly affect them as microconditions influencing their access to safety. These factors include the nurses' response to immunizations, which ranged from opposition to acceptance of vaccination. Other factors include the nurses' living conditions, how they commute to work, their personal-social characteristics, personality traits, underlying disease status, and their willingness to work in the COVID-19 unit.

*“I'm married and have two children. I also take care of the housework. I cooked for my family even when I was sick. With the heavy equipment we use, I'm constantly on my feet even while I'm working: from the time I arrive to the time I return home. I mean, I'm completely worn out and my body has become weak. I'm exhausted. This is the case with most of my colleagues who have children. On the other hand, we're more anxious and worried that our children might catch it.” (P 8)*

On the contrary, a nurse living alone shared a contrasting experience:

*“I reside alone, so I didn't notice many changes at home compared to my colleagues who live with family. However, when I fell ill, I faced significant challenges. Being alone, there was no one to provide care or assistance. I had to manage everything on my own, which made the situation extremely challenging. I felt a profound sense of isolation and lack of support during this difficult time.” (P 21)*

*(2) Shielding Catalysts Framework*. Participants' experiences revealed that alongside various hindering factors, certain enabling elements played a role in enhancing nurses' safety, albeit with a lesser impact. These elements collectively form the “Shielding Catalysts Framework,” encompassing two subcategories: “Encouraging Resilience” and “Organization Boost.”

*Encouraging Resilience*. Supportive feedback and positive reinforcement were vital in bolstering nurses' resilience and well-being. Participants highlighted the significance of receiving encouragement amid challenging circumstances, including support from family members showing empathy. Positive feedback from peers, friends, and the community served as a source of hope and motivation, fostering their ability to withstand stressors and maintain psychological safety.

*“My father used to say, “You must work under such circumstances.” He said, “For the education you have, you have to serve the people.” That added to my peace of mind, and I could bear the difficult situation better, but my husband was a little upset that I was working in the COVID ward.” (P 19)*

*Organization Boost*. The proactive stance of health authorities in implementing supportive measures reflects a commitment to fostering conducive work environments. Participants emphasized the significance of these initiatives, such as facilitating transfers for staff facing unique challenges, prioritizing vaccination for healthcare workers, and implementing motivational strategies. These efforts were instrumental in mitigating nurses' psychological stress and enhancing overall well-being.

*“Breastfeeding and pregnant nurses and those with health problems were isolated, and they didn't allow this group to stay in the COVID ward. That alone made my colleagues and me feel like they cared about our health and our children's health, which was a factor in nurse satisfaction.” (P 16)*

#### 3.1.2. Navigating Safety Threats

The contextual conditions identified as obstacles and catalysts shaped the nurses' perceptions of safety threats and challenges. These were represented by three categories and seven subcategories (refer to Table 2 and Figure 1). The categories, named “Transformations,” “Inequalities,” and “Emotional,” reflect the multifaceted nature of the issues faced by nurses in ensuring their safety.

*(1) Transformations*. Transformations refer to significant changes in the personal and professional lives of nurses due to the challenging working conditions brought on by the COVID-19 pandemic. The circumstances of nurses' personal and family lives have been dramatically altered.

*“There is no way to plan anything. Our education is interrupted. Our work and personal lives are in limbo. My whole life is on hold.” (P 13)*

In addition, there were both positive and negative changes at the professional level that impacted nurses' access to safety. The “Transformations” category encompasses changes in both personal life and professional transitions.

*Changes in Personal Life*. Participants stated that they had made changes in their daily lives since the beginning of the COVID-19 pandemic to ensure the highest level of safety. However, some of these changes led to excessive fatigue and vulnerability of their immune systems.

*“I change my clothes in the parking lot and hang them in the storage room. Then I go straight to the bathroom and sometimes shower twice a day. I was really scared when I contracted the virus, especially after the second infection.” (P 9)*

Some nurses mentioned that their attitude toward life has changed compared to before the pandemic triggering the capacity to see more meaning in their lives and a more positive outlook. They decided not to be overwhelmed by details, to value time with loved ones, and to seize the moment. This new attitude provided them with inner peace, reduced their fear of the virus, and possibly increased their psychological safety.

*“My attitude toward life has changed compared to the time before the pandemic. I owe this change to the coronavirus. Now I enjoy every moment I spend with my loved ones. I have decided to ignore trivial things because I have seen death up close. Trivial things should not destroy the value of being together. This change in my perspective has brought me peace.” (P 16)*

In many cases, the mentioned changes caused disruptions in the normal flow of personal and family life, also delaying or interrupting daily plans. At the family level, these changes were evident in restricted emotions, families' worries about the nurses' health, disapproval of the nurses' work in the COVID-19 ward, concerns about inadequate supply of PPE, and fear of getting infected and coming to the hospital. These factors, in turn, increased the psychological pressure on nurses.

*“In the first few months, I was going straight to my room when I was coming home from the hospital because I was afraid of infecting my family. Even my mother would put my food behind the door so I could pick it up later. I did not talk to my family at all, which was very worrying for both my family and me.” (P 1)*

*Professional Transitions*. Participants experienced difficult transitions at the professional level, including changes in performance and professional wellness triggering burnout. They also rediscovered their passion for the nursing profession. Nursing care was delivered with extreme caution, but poor nursing care caused by factors such as fear of being close to patients resulted in changes in nurses' performance. These changes posed threats to nurses' psychological safety, leading to psychological exhaustion and discomfort.

*“The workload was so heavy that there was no coordination among the staff, and most of them were rookies. The crowding and extreme pressure were exhausting. Sometimes I completely forgot when I had visited my patients, and my duties were limited to administering medications and I was not able to attend to the other needs of the patients, which put a lot of stress on me.” (P 17)*

The unfavorable working conditions, the heavy workloads, long and busy shifts, limited support, witnessing patients' pain, suffering, and death, and other issues caused nurses to experience severe fatigue, physical and mental trauma, sleep disturbances, nightmares, and a sense of helplessness.

*“The working conditions here are extremely difficult. You care for one patient, only to discover that another one requires your attention. Despite only being assigned two patients, the workload seems never-ending. Occasionally, due to staffing shortages, we are forced to care for three patients at once. This has drained our energy and left us lacking the motivation to carry on.” (P 19)*

In contrast, the media and public attention given to the nursing profession and nurses fostered a sense of worth and altruism, and ultimately rediscovered love for the profession among nurses.

*“The COVID-19 pandemic raised some awareness in society about nurses, the nursing profession, and its values and risks. It made us happy, motivated us, and eased the psychological burden for me and my colleagues. Therefore, I think the nursing community should be grateful for the COVID-19 pandemic, although it has suffered a lot.” (P 24)*

*(2) Inequalities*. During the pandemic, nurses experienced a variety of unfair treatments and discrimination within and between professions, negatively affecting their psychological, physical, and professional safety.

*Intraprofessional Inequalities*. Intraprofessional inequalities refer to the disparities between nurses within the same center or between nurses in different centers. Some participants pointed out that there was inequity in the allocation of sick leave among nurses with varying employment statuses. Additionally, there reported perceived inequities among nurses based on their years of experience working on the COVID-19 ward and being assigned to critically ill patients.

*“There was a difference between agency staff and permanent or contract nurses. For example, we weren't entitled to sick leave if we got infected. And they wouldn't pay for it if we left work. This made us frustrated and unmotivated.” (P 8)*

One participant described the impact of employment status on nurses' safety as follows:

*“After a few months, they refused to pay us because we were contract workers. Some of my friends and I had to work in two places to make ends meet. Now, this arrangement of working in two places exhausted us and weakened our immune systems. On the other hand, job insecurity was getting to us.” (P 22)*

Participants also mentioned that there were differences between nurses at different centers in various areas regarding PPE allocation and financial compensation, which caused psychological distress.

*“Well, I heard that the nurses in hospital X receive financial allowances in addition to full PPE kits, while we barely manage to ration our PPE. Why does it have to be this way? It added to the psychological burden for us.” (P 12)*

*Interprofessional Inequalities*. There were various forms of inequality among physicians and nurses in terms of PPE allocation, patient visitation protocols, and compensation, including the “COVID-19 bonus.” There were also disparities between nurses and administrative staff, as well as external organizational inequalities.

*“There was a difference between us and the physicians in terms of getting equipment. They easily got as many pieces as they requested, but for us, it was rationed. Even now, we get our N95 masks under our doctor's name in our station. This kind of treatment by the authorities was hurtful to us.” (P 10)*

The other level of inequity was between nurses and administrative staff in the form of diagnostic laboratory test accessibility.

*“Well, despite the protocol to do the PCR test for us, the nurses would compulsorily do the tests only in the case of presenting severe clinical symptoms in the almost-dying phase! But the administrators could easily test themselves once in a while. Well, that kind of thing puts psychological stress on you.” (P 11)*

Some nurses expressed feeling discriminated against when they observed administrators in other organizations readily approving sick leave and providing free PPE for their administrative colleagues. These factors worsened the psychological distress experienced by the nurses.

*“You know, compared to hospitals, other organizations care much more about the health of their employees. For example, I have a relative who works somewhere else. They get sick leave for even the mildest symptoms and get PPE not only for themselves but also for their family members…” (P 17)*

*(3) Emotional Challenges*. Emotional challenges included unpleasant emotional repercussions, the experience of reason-emotion dichotomy that arose from confronting phenomena that contradict professional values, and the experience of stigma associated with COVID-19.

*Unpleasant Emotional Repercussions*. Most participants experienced various unpleasant psychological effects, such as fear, terror, intense anxiety, and dread of an unknown future. As the pandemic lasted for a long time, they also underwent changes in psychological effects, such as experiencing a depressive and sad phase, weakening nurses' immune systems, and jeopardizing their safety.

*“For example, my colleagues used to talk about another year [of the pandemic crisis]. Then they would say to me, “Given how things are going, do you think you'll survive another year? I doubt it very much. … there's a kind of depressed mood.” (P 15)*

*Reason-Emotion Dichotomy*. Moral dilemmas arose from confronting double-sided phenomena that contradict professional values. The lack of facilities forced them to prioritize care, leading to anguish and problems for the nurses in the form of guilt.

*“We had a shortage of ventilators …. This was very stressful psychologically and led to additional fatigue.” (P 4)*

*Sadness of Stigma*. Sadness of stigma included negative behaviors and attitudes, labeling, avoidance, and rejection related to nurses, especially those in direct contact with COVID-19 patients. Nurses experienced various stigmas, from their families to their neighbors, colleagues, and the public. The perceived stigma posed a threat to the safety of the nurses.

*“The avoidance behavior of others was hurtful. But what did we do? We were taking care of patients in terrible conditions. At the same time, others, including some family members, friends, and people, annoyed us by giving us strange looks, avoiding us, and even saying nurses were sources of infection.” (P 21)*

#### 3.1.3. Strategies

Several strategies have been implemented by participant nurses represented by the “seeking protection” category, as articulated in four subcategories (Table 2 and Figure 1). These strategies implied changes in working lifestyle, inappropriate responses, efforts to reduce psychological stress, and providing mutual care. All of these strategies have been enacted to help nurses stay on track despite the challenges they face.

*(1) Change of Working Lifestyle*. Nurses employed several strategies to improve their work lifestyle. These strategies included taking preventive measures, regulating the arrival and departure of companions/visitors, issuing safety alerts, educating patients and their caregivers, conducting peer training, seeking help, obtaining information from credible sources, practicing self-care due to a lack of PPE, managing physical trauma, and lodging complaints to authorities regarding their workplace protection.

*“I would try to get information from clinical supervisors, doctors, or university professors about COVID, such as how to protect myself and control the disease.” (P 2)*

*Inappropriate Responses*. According to participants' experiences, the onset of the pandemic led to increased work pressure and fatigue, which resulted in the adoption of inappropriate responses. Some nurses displayed maladaptive responses to the COVID-19 crisis, such as aggression toward patients and companions, avoidance of certain situations, negative coping mechanisms, contact avoidance, obsessive protection, and secrecy. These responses were linked to negative outcomes, such as increased personal tension, strain within the organization, and potential harm to patients.

*“Some people's avoidance behavior made me conceal my work in the COVID ward. Whenever someone asked, I would deny that our center had cases of coronavirus. However, my conscience kept bothering me, reminding me that I was in close contact with patients and needed to ensure their immune systems were not compromised to get severe illness.” (P 17)*

*Psychological Stress Alleviation Strategies*. One strategy that significantly helped in reducing psychological stress and improving well-being was filtering incoming information, spending time with family and friends, turning to spirituality, engaging in recreational activities, fostering hope, creating a positive environment, desensitizing, seeking medical assistance, and trying to combat stigma.

*“When I take breaks, I listen to one of my favorite songs on my phone to lift my spirits.” (P 9)*

These strategies decreased psychological pressure, enhanced relationships, physical well-being and immunity, improved sleep quality, and better management of physical issues.

*“Since I started exercising and focusing more on spirituality, like reading the Quran before bed, my nightmares have ceased. I also experience less stress and fewer heart palpitations.” (P 21)*

*Mutual Care*. Mutual care, expressed in the form of asking for and giving family support, limiting interactions, being stricter about personal hygiene than ever before, practicing self-care, using alternative treatments, and supporting colleagues, was a strategy used by most participants due to their sense of responsibility for others. These strategies played an important role in restoring calm to the family and improving relationships. However, during the ongoing pandemic, the strategy of self-quarantine, which prevented the opportunity to give and receive mutual support, induced feelings of sadness.

*“During this time, we canceled all gatherings, funerals, and weddings, even visits to our parents, because I was afraid of being a carrier and infecting others. We haven't taken a trip in two years.” (P 20)*

#### 3.1.4. Outcomes

Outcomes of using the strategies mentioned above to protect oneself and others varied between two categories (Figure 1 and Table 2) as

1. “Bright horizon” indicates satisfaction (reduction of psychological stress, improvement of relationships, and restoration of family peace) and physical protection (immunity, reduction of sleep problems, and management of physical problems)
2. “Unpleasant reflection” indicates individual stress (increased likelihood of infection, physical complications, regret, feelings of neglect, and sadness), weakening of the organization (exacerbation of the lack of power, greater spread of disease, and a decrease in trust), and the risk of harm to patients (delivering poor quality of care, compromising patient safety, and being dissatisfied with patients and their relatives). However, with the increased scientific understanding of the COVID-19 disease nurses continued to seek protection in the heart of the storm but the contextual factors changed:

*“As time goes on, I feel that everyone has gotten over the shock, that scientific information is increasing, that training groups are being formed in the hospital, and that there is some comfort in hearing that the disease can be controlled if you increase your knowledge and follow the protocols.” (P 6)*

## 4. Discussion

The theory “seeking safety in the heart of the storm” explains how nurses navigate safety challenges during pandemics, incorporating concepts, such as transformations, inequalities, and emotional challenges. It highlights the balance between hopeful outlooks and sobering reflections, ultimately leading to the core concept of seeking protection in the heart of the storm. By drawing on established theories like coping, resilience, and social support, a comprehensive framework emerges to understand nurses' safety-seeking behaviors [24, 25]. Additionally, it aligns with Kang and colleagues' model (2023) of “seeking to protect the safety of oneself and the patient” to describe the experiences of nursing students facing safety-threatening conditions and their adaptation process [26]. Our theory compares well with that of Kang et al., addressing obstacles, positive and negative influencing factors, effective and ineffective strategies used by nurses to maintain protection, and resulting outcomes. As a result, it stands as a comprehensive theory for understanding the adaptive strategies of nurses during crises.

First, contextual factors emerged as influencing the protection of hospital nurses during the COVID-19 pandemic, shedding light on the obstacles and catalysts that shape their journey toward safety and protection. Among the organizational challenges, staffing, information gathering, and training [27], protection issues and unfavorable infrastructure factors emerged, as in previous studies [3, 28]. Additionally, inappropriate monitoring and supervision, lack of organizational support [29], and varying ward-related protection conditions [30] also emerged as already well-documented, indicating that nurses worldwide have faced similar organizational conditions. However, the time-consuming documentation appears to have played a significant role in Iran, suggesting that digital transformation may ease this burden in the future.

In terms of patient/relative-related challenges, they appear to be similar to those reported in previous studies [31]. The presence of patients and their families has been highlighted as crucial for nurses in alleviating distress [32]. Consequently, harassment, frequent calls and follow-ups, unreasonable expectations, and misconceptions among patients and relatives pose a serious threat to nurses' safety and health [33, 34]. Also, these findings are in line with those reported in previous studies. However, noncompliance with restrictions or preventive measures was also reported, possibly due to the emotional bonding in Iranian culture between family members and patients' relatives demanding the lifting of visiting restrictions. Moreover, nursing-related factors have also been documented in previous studies, such as reactions to vaccination [35], living conditions [36], transportation to work [37], personality [38], underlying disease [39], and willingness to visit the COVID ward.

The experiences of nurses during the pandemic showed a spectrum of psychological reactions. Initially, nurses were confronted with negative feelings, such as anxiety, fear, shock, and stress. Over time, they adapted to the new working conditions, but also showed symptoms of depression, a sense of hopelessness in the face of the never-ending situation, and occupational burnout due to the ongoing challenges of the pandemic [28, 36].

The shielding catalysts framework acted as a guiding light for nurses navigating the path to protection during the COVID-19 pandemic, incorporating elements, such as fostering resilience and enhancing organizational support. Societal and family support played a crucial role in enduring difficult conditions and enhancing self-esteem, and readiness for work [36, 40]. Various forms of support, such as providing information, extending work shift intervals, offering childcare facilities for nurses' children, and mental support, greatly contributed to nurses feeling secure [36].

Nurses' perceptions of safety and protection threats and challenges were influenced by contextual conditions. These influences gave rise to three main lived experiences, each closely tied to concerns about safety and protection: “transformations,” “inequalities,” and “emotional challenges.” The first refers to forced and deliberate changes in personal life [31, 41] requiring modifications to achieve peace of mind, such as showing more appreciation for life and spending time with family as a form of “psychological growth” [36]. Moreover, changes at the professional level were also reported: a different mode of care was delivered in terms of human interaction and physical contact with patients [42] requiring professional changes in the performance and limiting the nurses' presence in the patient room, i.e., due to the fear of contracting the virus, the need to reduce physical contact with patients [43], and PPE deficiencies [36]. Occupational burnout and the negative experience in these transformations seem to be contrasted by the nurses' sense of competence, accomplishment [40], and pride [31], which may have increased the professional commitment, satisfaction, and intention to continue to work as a nurse [44].

Inequalities are another emerged core category: discrimination has been reported as increasing during the COVID-19 pandemic [45], leading to different reactions that negatively affect both professional engagement and performance. Nurses' moral dilemmas have also increased, as reported elsewhere [46]. Despite their efforts to save lives, nurses have often been considered a source of viral transmission during the COVID-19 pandemic [40]. A lack of adequate awareness in society is one of the main reasons for occupational stigma [47], and providing sufficient information may help to reduce it [48]. Overall, the emotional challenges that result may have triggered the intention to leave the profession [49], a phenomenon largely documented in the postpandemic era.

“Seeking protection” was the term used to describe the strategies employed by nurses to protect themselves and others. “Changing the working lifestyle” involved adhering to preventive measures in the wards, managing lockdowns, and safety alerts; seeking help and training; obtaining information from credible sources; self-managing PPE shortages; and complaining to authorities about the need for protection in the workplace, as documented in previous studies [28, 40]. However, in their efforts to safeguard themselves, nurses resorted to inappropriate strategies, such as avoidance (of situations or contacts), aggression (toward patients or relatives), or obsession (such as excessive searching for protection). Avoidant behavior was reported in studies involving nurses during the Ebola outbreak [50], while compulsive behavior in following protocols was reported by nurses in Turkey and Qatar [40, 51]. This ineffective behavior may further escalate feelings of being unsafe and negatively impact the work environment climate and personal well-being of nurses.

In applying “Psychological Stress Alleviation Strategies,” several actions undertaken have already been documented. Examples include filtering incoming information, interacting with family and friends, resorting to spirituality and leisure activities, fostering a sense of hope, creating a joyful atmosphere, seeking medical help [52], and attempting to combat stigma by concealing facts, limiting relationships, and following protocols more strictly [40, 51, 53]. Providing “mutual care” to protect themselves and others, self-imposing intense isolation [31, 36, 43], strictly adhering to protective protocols, and the fear of losing relatives have also been documented [54]. The support and help that colleagues provide to each other using compassion and collaboration techniques in the workplace ease the workloads and enhance nurses' sense of safety [36, 40]. Discussions with colleagues and receiving updates and information from managers who listened and responded to feedback have been highlighted as being more helpful than external support mechanisms [55].

The perceived outcome varied between “Bright horizon” and “Unpleasant reflections.” The former was reported in other studies, such as in a protective feeling among caregivers after vaccination [56], and increased resilience and peace of mind due to trust in God and the use of self-care strategies [52, 57]. On the other hand, the latter can result in overlooking the role of family members in the care process [29, 53] or further compromising the safety and quality of care [30, 58].

### 4.1. Limitations

Our study has several limitations. First, due to the qualitative nature of the study, our findings may not be generalizable to other communities or contexts. Second, some interviews were conducted via video calls based on participant preferences, potentially limiting the depth of shared experiences. Third, while the extended study period (2020 to 2022) allowed for the development of a theory encompassing all pandemic stages, its validity requires confirmation across different cultures, countries, and pandemic waves. Fourth, the study focused solely on hospital nurses; therefore, the theory should be validated in various settings, disciplines, and professions to achieve a more comprehensive understanding of the phenomenon, which could impact all healthcare professionals.

## 5. Conclusions

To the best of our knowledge, despite numerous studies on nurses' perceived safety during the recent pandemic, a comprehensive grounded theory describing the process of seeking protection by frontline nurses has not been formulated to date. With this qualitative inquiry, we attempted to address this gap by delving into how hospital nurses sought protection during COVID-19.

At an overall level, the study developed the “seeking protection in the heart of the storm” theory, illustrating nurses' efforts during the pandemic. Available literature has already produced evidence; however, the fragmented evidence has not been included in a comprehensive picture. Nurses faced various challenges, termed “Safety Pathway Obstacles,” including organizational, patient-related, temporal, and nurse-specific issues. These challenges coalesced into three themes: Transformations, Inequalities, and Emotional Challenges, emphasizing the complexities of safety in nursing care. Despite these obstacles, nurses adopted protective measures, implying lifestyle adjustments and coping strategies, resulting in both positive outcomes (satisfaction, well-being) and negative outcomes (stress, patient harm). Taken together, the results highlight the intricate interplay of adversity, resilience, and practices adopted by nurses during difficult times. Findings provide a composite picture depicting nurses striving to achieve safety to shield themselves and their families from the relentless impact of the disease. This imperative accentuated their awareness and dedication to navigating toward safety, prompting the adoption of diverse strategies, some effective and others not, propelled by the urgent need to secure safety. These choices significantly influenced outcomes, leading to either positive or negative consequences. In essence, the research sheds light on the intricate nature of nurses' experiences and underscores the crucial role of nurturing supportive work environments and policies to enable effective coping mechanisms. By acknowledging the challenges confronted and the strategies employed by nurses, healthcare institutions can offer better support to frontline workers, thereby improving overall patient care outcomes. These elements can inform informative, educational, and organizational strategies at the local, regional, and national levels, aiming to capitalize on the lived experience of nurses in a proactive strategy to protect them during difficult times.

## 6. Implications for Nursing Management

Developing a theory can enhance our understanding of phenomena informing customized preventive measures and evaluating their effectiveness. First and foremost, there is an urgent need for a cohesive strategy to protect nurses, acknowledging their safety as a public health priority. Additionally, investments in cultural, educational, and organizational resources should prioritize nursing roles at both macro- and microlevels. Third, promoting individual preparedness is crucial. It is important to acknowledge that not all strategies employed by nurses yield positive outcomes, emphasizing the importance of effective coping strategies.

Moreover, researchers should evaluate the theory's applicability across different contexts and cultures to ensure its empirical validity. Policymakers, managers, and educators can use the emerged insights to inform decision-making and educational initiatives according to nurses' concerns and coping mechanisms. In clinical settings, healthcare professionals, especially nurses, can learn from successful strategies to enhance care provision during crises. Embedding process of seeking protection into nursing education and healthcare provider training programs can further enhance preparedness.

## Figures and Tables

**Table 1:** Participants' main characteristics.

Nurse ID^§^	Age^¶^	Gender	Marital status	Education	Employment status	Ward	Experience (years)	In COVID-19 ward (months)
1	35–39	Female	Single	MNSc^‡^ student	Permanent civil employment	Critical COVID-19	13	12
2	40–44	Female	Single	BNSc^†^	Contract employment	Critical COVID-19	17	12
3	25–29	Male	Single	BNSc	Permanent civil employment	COVID-19 and non-COVID-19	5	12
4	25–29	Male	Married	BNSc	Contract employment	Critical COVID-19	2	12
5	35–39	Female	Single	BNSc	Permanent civil employment	Non-COVID-19	6	–
6	40–44	Female	Single	MNSc	Permanent civil employment	Critical COVID-19	17	5
7	30–34	Female	Married	MNSc student	Trainee nurse	COVID-19 and non-COVID-19	1	5
8	35–39	Female	Married	BNSc	Contract employment	COVID-19 and non-COVID-19	11	5
9	30–34	Female	Married	BNSc	Contract employment	Critical COVID-19	8	5
10	25–29	Female	Married	BNSc	Contract employment	Critical COVID-19	5	5
11	35–39	Female	Married	BNSc	Permanent civil employment	Critical COVID-19	17	5
12	25–29	Male	Single	MNSc student	Contract employment	COVID-19 and non-COVID-19	4	6
13	35–39	Male	Married	BNSc	Permanent civil employment	Critical COVID-19	10	8
14	30–34	Male	Married	MNSc	Permanent civil employment	Critical COVID-19	8	5
15	25–29	Male	Single	BNSc	Permanent civil employment	Critical COVID-19	4	3
16	35–39	Female	Married	MNSc	Permanent civil employment	Critical COVID-19	11	12
17	25–29	Female	Single	BNSc	Contract employment	Critical COVID-19	5	10
18	45–49	Female	Single	BNSc	Permanent civil employment	Critical COVID-19	20	1
19	25–29	Female	Married	MNSc	Permanent civil employment	COVID-19 and non-COVID-19	2	12
20	40–44	Female	Divorced	BNSc	Permanent civil employment	Wound specialist	13	20
21	25–29	Male	Single	BNSc	Contract employment	COVID-19 and non-COVID-19	6	20
22	30–34	Male	Single	BNSc	Contract employment	COVID-19 and non-COVID-19	6	20
23	45–49	Female	Married	BNSc	Permanent civil employment	Esophageal echocardiography	14	–
24	40–44	Female	Married	BNSc	Permanent civil employment	Angiography	18	4
25	45–49	Female	Married	MNSc	Permanent civil employment	Clinical supervisor	24	20

^§^Identity code; ^¶^Age was provided in ranges to ensure anonymity; ^†^Bachelor of Nursing Science; ^‡^Master of Nursing Science.

**Table 2:** The process of “seeking protection in the heart of the storm” among hospital nurses during the COVID-19 pandemic.

Paradigm components	Categories	Subcategories
Contextual conditions	Safety pathway obstacles	Organizational challenges
		Patient/relative-related challenges
		Time-related challenges
		Nurse-related challenges
	Shielding catalysts framework	Encouraging resilience
		Organization boost

Navigating safety threats	Transformations	Changes in personal life
		Professional transitions
	Inequalities	Intraprofessional inequalities
		Interprofessional inequalities
	Emotions	Unpleasant emotional repercussions
		Reason-emotion dichotomy
		Sadness of stigma

Strategies	Seeking protection	Change of working lifestyle
		Inappropriate responses
		Psychological stress alleviation strategies
		Mutual care

Outcomes	Bright horizon	Satisfaction
		Physical protection
	Unpleasant reflection	Individual stress
		Organizational vulnerability
		Patient injury

## Data Availability

The data supporting the findings are available from the corresponding author. The data are not publicly available due to privacy or ethical restrictions.

## Supplementary Materials

Supplementary Materials Standards for Reporting Qualitative Research (SRQR) and an example of the process of forming a main category as a supplementary material file will be submitted with our manuscript.
