# Supplementary material for: Seeking Protection in the Heart of the Storm: Findings from a Grounded Theory Study
Source: J Nurs Manag. 2024 Jul 30;2024:6185455. doi: 10.1155/2024/6185455 (PMC11918800; doi:10.1155/2024/6185455)
Supplement: Supplementary Materials — Standards for Reporting Qualitative Research (SRQR) and an example of the process of forming a main category as a supplementary material file will be submitted with our manuscript. [file 6185455.f1.zip › Supplementary Table 2docxrevised_ (2).docx]

| Supplementary Table 2. An example of the process of formation of a main category | | | |
| --- | --- | --- | --- |
| Main Category | Subcategories  Actions and processes | Codes | Examples of Quotes |
| Transformations | Changes in personal life | Worrying about the unknown and uncertain future, worrying about interrupting and delaying personal and academic life, dropping out of normal life, changing one's whole life, not being able to plan because of the difficulty of the situation, not going to the hairdresser for fear of being a carrier and/or getting infected, not hiring a caregiver for the children because of fear of being a carrier for the caregiver, assigning and keeping separate clothes for the workplace, changing work clothes outside the home, washing hands a lot, washing and disinfecting all equipment, increasing the frequency of bathing and washing clothes,  Making the most of life's moments, considering it important to be with loved ones, feeling calm by changing the way you look at life, realizing the meaning of life, the difficulty of following the restrictions at home, the family's fear and concern that the nurse might be infected and be a carrier, the family's discomfort, and resistance to working in the Corona ward, the family's concern for the nurse's health, the friends' concern for the nurse's health, the family's concern for the infection and hospitalization of colleagues, family concern about the adequacy of PPE**, spouse's failure to take illness seriously, discomfort due to family illness or death, unwillingness to be hospitalized despite severe lung involvement because of fear that parents may become infected, guilt due to possibility of family being carriers, staying away from family during quarantine, lack of time and reduced emotional ties with family, intolerance of being away from family. | *“There is no way to plan anything. Our education is interrupted. Our work and personal lives are in limbo. My whole life is on hold.” (Participant 13)*  *“I change my clothes in the parking lot and hang them in the storage room. Then I go straight to the bathroom and sometimes shower twice a day. I was really scared when I contracted the virus, especially after the second infection.” (Participant 9)*  *“My attitude toward life has changed compared to the time before the pandemic. I owe this change to the coronavirus. Now I enjoy every moment I spend with my loved ones. I have decided to ignore trivial things because I have seen death up close. Trivial things should not destroy the value of being together. This change in my perspective has brought me peace.” (Participant 16)*  *“When I come home, I am not allowed to take my dirty clothes home, my relationships with my children have become more limited, for example, hugging and kissing them has decreased, and I feel very sad because of that.” (Participant 11)*  *“In the first few months, I was going straight to my room when I was coming home from the hospital because I was afraid of infecting my family. Even my mother would put my food behind the door so I could pick it up later. I did not talk to my family at all, which was very worrying for both my family and me.” (Participant 1)*  *“The family did not like that I worked in the Corona department, especially my husband did not like that I worked. When they heard that our department became the Corona department, they resisted and said we should change departments, but we stayed and worked.” (Participant 17)* |
|  | Professional transitions | Fear of caring for a positive patient in a non-Covid department, high mortality due to poor volunteer efficiency, inadequate care due to shift compaction and heavy workload, decreased performance due to fear, slow and difficult performance with PPE, difficulty walking and shaking due to overprotectiveness, inability of nurses to provide comprehensive care including psychological care because of lack of contact with patients, discomfort because of neglecting patients due to workload and high documentation, discomfort because of inability to communicate with patients due to high workload, energy depletion of nurses with the prolongation of the pandemic, the occurrence of death beyond the control of nurses, psychological pressure due to the high mortality rate, discomfort related to the crying of companions when patients die, discomfort due to the intubation of patients because of the low probability of recovery, discomfort due to the severe lung involvement and death of young patients, discomfort due to death of patients because of delayed visits, discomfort at seeing the pain and suffering of patients, high pressure due to high rotation of staff, discomfort due to prolonged illness, discomfort due to uncertainty and conditions such as war, heavy and unpleasant atmosphere when caring for the families of colleagues, crowded shifts, factors of psychological pressure, feeling more pressure and excitement with the start of accreditation documentation, decreasing motivation due to excessive involvement of people in nurses' work, discomfort due to providing too much information to people, and decreasing confidence and increasing fear of not doing anything about physical problems due to fatigue. difficulty not having time to complain about problems due to high work pressure, difficulty tolerating PPE long term, belief in infection due to daily exposure, infection due to insufficient rest, belief in higher likelihood of infection due to fatigue and weakening of immune system, belief in weakening of immune system due to lack of rest, illness of colleagues due to shift compaction and lack of rest, increase in illness of coworkers due to continuation of illness, the feeling of value at the community level due to being part of the medical staff, society's appreciation of nurses, noting the value of nurses' work, people's appreciation of nurses' commitment, improvement in physicians' view of nurses, the relative improvement in people's view of nurses, the feeling of strength of heart due to the spread of nurses' hard work from More recognition of nursing in the era of Corona, clarification of the role of nursing in society, the joy of presenting a better image of nursing, visibility of the face of nursing, comprehensive care of patients despite the possibility of infection, dealing with patients with a cheerful spirit, sense of fulfillment of duty in these difficult conditions, increasing experience in nursing, remaining in the profession because of society's need for a nursing profession, having no financial motivation to work, helping one's fellow man, love and passion to help people | *“In the beginning, when we had a patient with suspected corona virus on the no corona ward, we were so concerned that we put the patient in a separate room, closed the door to his room, and gave the patient medications through the back door. But then we all felt guilty for locking the poor patient in the room and even worse for scaring him.” (Participant 7)*  *“The hustle and bustle of the shifts, changing clothes every shift, and the discomfort of the patients being overworked made us very tired, so these factors weakened our immune system and we got sick more often.” (Participant 22)*  *“Working under these harsh conditions with a heavy workload caused us many physical problems. I went to the doctor myself when I first got sick. They said I had hyperthyroidism. I always checked my tests. I did not have any problems before that.” (Participant 14)*  *“The workload was so heavy that there was no coordination among the staff, and most of them were rookies. The crowding and extreme pressure were exhausting. Sometimes I completely forgot when I had visited my patients, and my duties were limited to administering medications and I was not able to attend to the other needs of the patients, which put a lot of stress on me.” (Participant 17).*  *“The working conditions here are extremely hard. You take care of one patient, only to find that another one needs you. We only work with two patients, and yet there is no end to the work. Sometimes, when there is a staff shortage, we have to work with three patients. This has sapped our energy, and we no longer have the drive to continue.” (Participant 19).*  *"We take care of a patient for a month or at least 20 days, and then the person you thought was a COVID-19* patient is intubated. It's very difficult to extubate him/her, plus it would affect the bad person's mood, you said. I work, hey, every day we consume energy, we change the history of the past, every day, send this test, that test, well, these useless things have consumed our energy." (Participant 18)*  *“This is very interesting. I once went to buy fruit from a delivery lorry. I do not know what happened. He found out I was a nurse. He took the fruit away from me. He went back and picked out the fresh fruit himself and put it in my car. He said that you, the nurses, are responsible for us. Getting answers Positive thoughts from others would drive away all my tiredness and I would start my work with energy.” (Participant 20)*  *“The pandemic COVID-19 raised some awareness in society about nurses, the nursing profession, and its values and risks. It made us happy, motivated us, and eased the psychological burden for me and my colleagues. Therefore, I think the nursing community should be grateful for the COVID-19 pandemic, although it has suffered a lot.” (Participant 24)*  *“With all the hardships, anxiety, and stress that many of my colleagues and I have had and that has increased, but because people need us now, we cannot let go because society needs the nursing profession and with all the problems we have, we try not to neglect the care of the patients. All of these factors had a positive effect on increasing mental calm and motivation to keep working.” (Participant 5)* |

*Coronavirus disease 2019 (COVID-19); **Personal protective equipment (PPE)
